# Network Localisation of White Matter Damage in Cerebral Small Vessel Disease

**DOI:** 10.1038/s41598-020-66013-w

**Published:** 2020-06-08

**Authors:** Marvin Petersen, Benedikt M. Frey, Eckhard Schlemm, Carola Mayer, Uta Hanning, Kristin Engelke, Jens Fiehler, Katrin Borof, Annika Jagodzinski, Christian Gerloff, Götz Thomalla, Bastian Cheng

**Affiliations:** 1 Department of Neurology, University Medical Center Hamburg-Eppendorf, Hamburg, Germany; 2 Department of Diagnostic and Interventional Neuroradiology, University Medical Center Hamburg-Eppendorf, Hamburg, Germany; 3 Epidemiological study center, University Medical Center Hamburg-Eppendorf, Hamburg, Germany; 4 Department of General and Interventional Cardiology, University Heart and Vascular Center, Hamburg, Germany

**Keywords:** Cerebrovascular disorders, Neurology, Neurological disorders, White matter disease

## Abstract

Cerebral small vessel disease (CSVD) is a widespread condition associated to stroke, dementia and depression. To shed light on its opaque pathophysiology, we conducted a neuroimaging study aiming to assess the location of CSVD-induced damage in the human brain network. Structural connectomes of 930 subjects of the Hamburg City Health Study were reconstructed from diffusion weighted imaging. The connectome edges were partitioned into groups according to specific schemes: (1) connection to grey matter regions, (2) course and length of underlying streamlines. Peak-width of skeletonised mean diffusivity (PSMD) - a surrogate marker for CSVD - was related to each edge group’s connectivity in a linear regression analysis allowing localisation of CSVD-induced effects. PSMD was associated with statistically significant decreases in connectivity of most investigated edge groups except those involved in connecting limbic, insular, temporal or cerebellar regions. Connectivity of interhemispheric and long intrahemispheric edges as well as edges connecting subcortical and frontal brain regions decreased most severely with increasing PSMD. In conclusion, MRI findings of CSVD are associated with widespread impairment of structural brain network connectivity, which supports the understanding of CSVD as a global brain disease. The pattern of regional preference might provide a link to clinical phenotypes of CSVD.

## Introduction

Cerebral small vessel disease (CSVD) is a condition comprising clinical, histopathological and imaging features thought to arise from damage to small perforating brain vessels^1^. Imaging markers considered to be manifestations of CSVD are recent small subcortical infarcts, lacunes, white matter hyperintensities of presumed vascular origin (WMH), enlarged perivascular spaces and cerebral microbleeds. The clinical sequelae of CSVD constitute the condition’s major relevance in the ageing western societies. As per current research, CSVD is associated with ischaemic and haemorrhagic stroke, cognitive decline, dementia, late-life depression as well as gait and urinary complaints^2–5^. However, open questions remain concerning the underlying pathophysiology and causal relations between CSVD and its clinical sequelae^6^.

Neuroimaging techniques are at the forefront of modern investigations of brain diseases. They offer insights into pathophysiological characteristics of various neurological disorders and thus also provide valuable information about the link between CSVD and its sequelae. On T2-weighted MRI data, WMH are a typical manifestation, however, brain tissue damage in CSVD is known to extend beyond lesions detectable by visual analysis. Diffusion weighted and diffusion tensor magnetic resonance imaging (DWI and DTI) in particular is capable of detecting CSVD-induced microstructural brain changes not visible on conventional T2-weighted data. Specifically, patients with CSVD were found to have altered microstructural properties in lesional and perilesional tissue detectable by DWI and DTI^7–9^. Based on these findings, a surrogate marker for the extent of CSVD termed “peak-width of skeletonised mean diffusivity” (PSMD) was proposed to capture microstructural white matter damage beyond traditional markers such as the volume of WMH. Therefore, PSMD features superior quantification capabilities while being robustly computable compared to other common CSVD surrogate markers^10^.

Connectomes are the subject of investigation in structural brain network analysis. They represent DWI-based reconstructions of human brain networks composed of nodes and internodal connections called edges^11^. In terms of anatomical correlates, nodes refer to cortical or subcortical grey matter areas, whereas edges represent the interconnecting white matter fibre tracts. Connectomes can be analysed by mathematical models to assess the integrity of brain networks *in vivo* and thus enable the appreciation of structural alterations in neurological disorders. In CSVD, application of graph theoretical measures to connectome data revealed decreased global efficiency of information transfer mediating cognitive symptoms^12^. While this finding describes pathological changes in large-scale brain network topology, connectome analysis also allows for localisation of altered structural integrity beyond the analysis of topological network parameters. In this study, we aimed to specify the impact of CSVD on the structural brain architecture using PSMD as a surrogate marker for CSVD. We chose to focus on two major aspects of white matter fibre tracts represented by edges in the structural connectome: first, edges were grouped according to interconnected grey matter areas to localise changes in distant yet connected brain regions. Second, edges were analysed according to their course and length (short or long intrahemispheric, interhemispheric) to specify the individual vulnerability of underlying white matter tracts. We hypothesised that structural disintegration relating to CSVD would affect a wide range of edges in the connectome. These changes would, however, show predominance for brain areas known to be involved in brain functions impaired in patients with CSVD.

## Methods

### Study population – the Hamburg City Health Study

The Hamburg City Health Study (HCHS) is a single center prospective, epidemiologic cohort study with emphasis on imaging to improve the identification of individuals at risk for major chronic diseases and to improve early diagnosis and survival. A detailed description of the overall study design has been published separately^13^. In brief, 45,000 citizens of the city of Hamburg, Germany, between 45 and 74 years are invited to an extensive baseline evaluation. A subgroup with present cardiovascular risk factors undergoes standardised MRI brain imaging. For this study, we analysed the first 1,000 brain MRI datasets from the HCHS baseline visit.

### Standard protocol approvals, registration and participants consents

The local ethics committee of the Landesärztekammer Hamburg (State of Hamburg Chamber of Medical Practitioners, PV5131) approved the HCHS and written informed consent was obtained from all participants. Good Clinical Practice (GCP), Good Epidemiological Practice (GEP) and the Declaration of Helsinki were the ethical guidelines that governed conduct of the study.

### MRI acquisition

Images were acquired using a 3-T Siemens Skyra MRI scanner (Siemens, Erlangen, Germany). Measurements were performed adapting a protocol as described previously^14^. In detail, for single-shell diffusion weighted imaging (DWI), 75 axial slices were obtained covering the whole brain with gradients (b = 1000 s/mm^2^) applied along 64 noncollinear directions with the following sequence parameters: repetition time (TR) = 8500 ms, echo time (TE) = 75 ms, slice thickness (ST) = 2 mm, in-plane resolution (IPR) = 2 × 2 mm, anterior-posterior phase-encoding direction. For 3D T1-weighted anatomical images, rapid acquisition gradient-echo sequence (MPRAGE) was used with the following sequence parameters: TR = 2500 ms, TE = 2.12 ms, 256 axial slices, ST = 0.94 mm, and IPR = 0.83 × 0.83 mm. 3D T2-weighted fluid attenuated inversion recovery (FLAIR) images were measured with the following sequence parameters: TR = 4700 ms, TE = 392 ms, 192 axial slices, ST = 0.9 mm, and IPR = 0.75 × 0.75 mm.

### Data preprocessing, connectome reconstruction and measurement of connectivity

All data was pre-processed using MRtrix 3.0 (http://www.mrtrix.org), Advanced Normalization Tools (ANTs, https://github.com/ANTsX/ANTs), the FMRIB Software Library 5.0.10 (FSL, https://fsl.fmrib.ox.ac.uk) and FreeSurfer 6.0 (https://surfer.nmr.mgh.harvard.edu)^15^. Preprocessing steps for connectome reconstruction included bias correction, brain extraction, parcellation via FreeSurfer and tractography based on constrained spherical deconvolution (CSD) with subsequent application of spherical-deconvolution informed filtering of tractograms (SIFT2)^16,17^. The detailed pipeline can be found in the supplementary materials. Network nodes were defined by parcellation of the grey matter areas in T1-weighted images according to the Desikan-Killiany atlas^18^. Connectomes were not thresholded prior to edge connectivity quantification^19^. Two nodes were assumed to be connected by an edge if DWI signal-derived streamlines were running between them.

**Figure 1:**
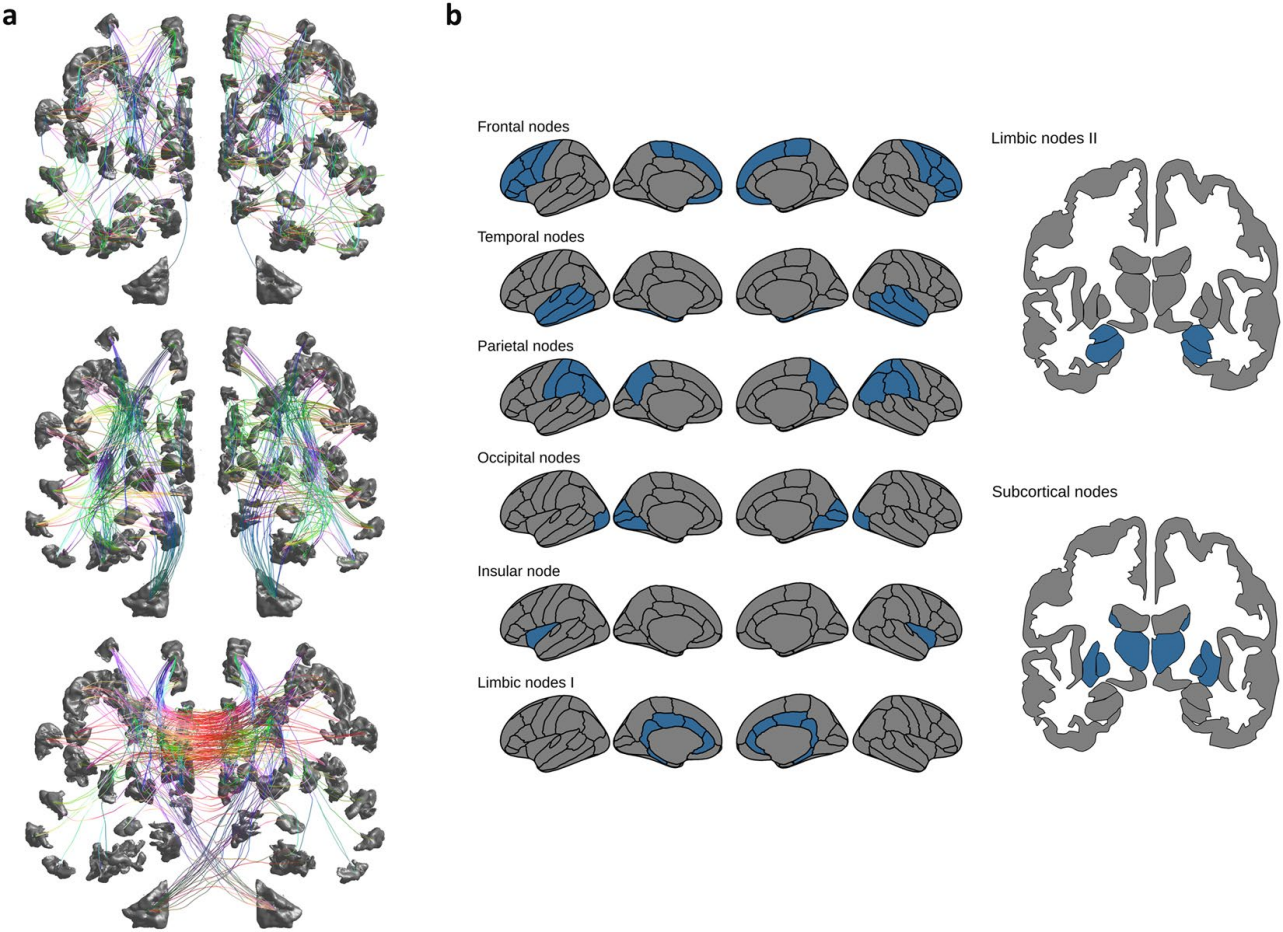
Illustration of edge grouping schemes applied in our study. Edges were grouped based on anatomical principles to investigate distinct impact of CSVD on different aspects of the human brain architecture. In **a**, the grouping by hemispheric course and length of edges is exemplarily illustrated from an anterior point of view. Grey areas represent nodes defined by atlas regions, edges are shown in colour coded by directional trajectory of white matter fibre tracts (X anterior-posterior, Y left-right, Z inferior superior). From top to bottom, intrahemispheric short edges, intrahemispheric long edges and interhemispheric edges are shown. In **b**, anatomical parcellations (grey areas) based on the Desikan atlas are shown. Edges were grouped by connectivity to a condensed selection of brain areas (blue). Abbreviations: CSVD = cerebral small vessel disease.

We investigated and localised the impact on white matter integrity in different components of the structural connectome. Therefore, network edges were grouped into categories representing two general anatomical aspects of brain architecture. Firstly, edges were grouped according to their connection to cortical brain regions using a predefined anatomical atlas that was condensed into major sections of the cerebral cortex, specifically frontal, temporal, parietal, occipital, limbic, insular, subcortical and cerebellar grey matter (Fig. 1b and Supplementary Table S1)^20^. Every edge was then assigned to these two regions based on which it connects. Thus, every edge was assigned twice: For example, an edge connecting the occipital and frontal region was allocated to both regions also meaning that an edge could count to the same group twice if it connected two regions from that group. Secondly, edges were grouped based on their length and course inside or between the two hemispheres (short or long intrahemispheric, interhemispheric) as illustrated in Fig. 1. Edges were determined as either running inter- or intrahemispherically, according to whether they were connecting brain regions on the same or different hemispheres, respectively. For definition of edge length, median streamline length of all edges was determined and their median was applied as a cut-off value for streamline length in each participant individually assigning edges into categories of short (if equal or below median) and long (if above median) edges. These definitions de facto resulted in three categories of network edges: long interhemispheric, long intrahemispheric and short intrahemispheric (see Fig. 1a)

The individual edge connectivity was determined by summing up the weights of streamlines reaching from one node to the other with higher sums indicating increased connection strength, i. e., higher connectivity. Due to the application of SIFT2 the connectivity of an edge corresponds to the density of underlying fibres as estimated by CSD and is therefore interpreted by us as an indirect measure of white matter integrity^17^. Connectivity values were then summed up for each group of edges with respect to localisation (i. e., frontal or temporal edges) and course (i. e., long or short, intra- or interhemispheric edges). Again, higher values indicate increased connection strength in these separate groups of edges, whereas lower values point to decreases in connection strength. In addition, relative connectivity was computed by dividing an edge group’s connectivity by the total connectivity of all edge groups.

### Peak-width of skeletonised mean diffusivity computation

The PSMD tool provided at http://www.psmd-marker.com was used to compute PSMD^10^. The process consists of two steps. First, DTI data including mean diffusivity (MD) were skeletonised via Tract Based Spatial Statistics Procedure (TBSS)^21^. PSMD is the difference between the 95th and 5th percentiles of the MD voxel values within the skeleton.

### White matter hyperintensity segmentation

We segmented WMH using the Brain Intensity AbNormality Classification Algorithm (BIANCA) implemented in FSL^22^. The training dataset consisted of masks of 100 participants and obtained by selecting only the voxels that were identified as WMH by two raters (MP, CM) independently via manual segmentation. White matter hyperintensity volumes were calculated.

### Statistical analysis

Linear regression models were applied to assess associations between values of PSMD as a surrogate marker of CSVD and an edge group’s connectivity. To account for the effects of age, sex and brain volume they were added as covariates to the linear models. Details regarding simple and multivariable linear models including actual p-values are listed in the supplementary materials (Supplementary Tables S2–S5). A formal interaction analysis served to ascertain the distinctiveness of the linear models (“connectivity ~ PSMD * fibre length group + (1|Subject)”, “connectivity ~ PSMD * grey matter area + (1|Subject)”, Supplementary Table S6). In addition, we calculated separate multivariable linear models including WMH load as defined as the volume of WMH normalised by individual brain volumes (Supplementary Tables S7 and S8). Statistical significance was defined as p < 0.05 and all p-values reported were corrected for multiple testing according to Bonferroni. Descriptive statistics of epidemiological and clinical data from all participants are provided as median and the interquartile range. The statistical analysis was performed in R (v3.1.4).

## Results

### Sample characteristics

In total, data from 1000 participants was available. After quality assessment of imaging data, 70 participants were excluded due to missing data (n = 21), poor quality or incompleteness (n = 40), or failed post-processing due to data incompatibility (n = 9). Thus, the analysis population encompassed 930 subjects. Epidemiological, clinical and imaging characteristics of all participants are shown in Table 1. Median age was 64 years (IQR = 14) and 45.6% of participants were female. Vascular risk factors were present in a considerable proportion of participants: arterial hypertension was present in 18.2% of participants, diabetes mellitus in 8.0% and 18.0% of participants were currently smoking.

**Table 1:** Sample characteristics and image analysis results - the Hamburg City Health Study.

Female sex [n, (%)]	424 (45.6%)
Age [years], median (IQR)	64 (14)
Vascular risk factors	
Current smoking, [n, (%)]	167 (18.0%)
Hypertension (>= 140/90 mm/Hg), [n, (%)]	169 (18.2%)
Diabetes, [n, (%)]	74 (8.0%)
Conventional MRI measures	
Brain volume [ml], median (IQR)	1483.7 (203.1)
WMH volume [ml], median (IQR)	0.6 (1.4)
WMH load [%], median (IQR)	4.4 (9.8)
Diffusion imaging measures	
PSMD, median (IQR)	0.0002 (0.0001)
Connectome densitiy [%], median (IQR)	88 (3)

Abbreviations: mm = millimeter, PSMD = peak-width of skeletonised mean diffusivity, WMH = white matter hyperintensities.

**Figure 2:**
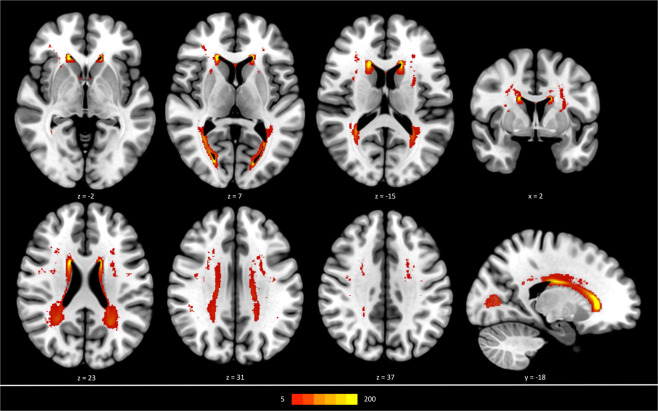
Distribution of white matter hyperintensities (in all participants, projected on a brain template in MNI-space. Frequency of WMH is illustrated as indicated by the colour bar. Z-values and Y-values refer to axial and sagittal slice position in MNI-space, respectively. Abbreviations: WMH = white matter hyperintensities.

Connectomes exhibited a median network density of 88% (IQR = 3%). Overall, the study sample showed only a minor to moderate lesion load with mainly periventricular distribution of WMH (see Fig. 2).

### Connectivity analysis

#### CSVD effects on grey matter area-specific connectivity

**Figure 3:**
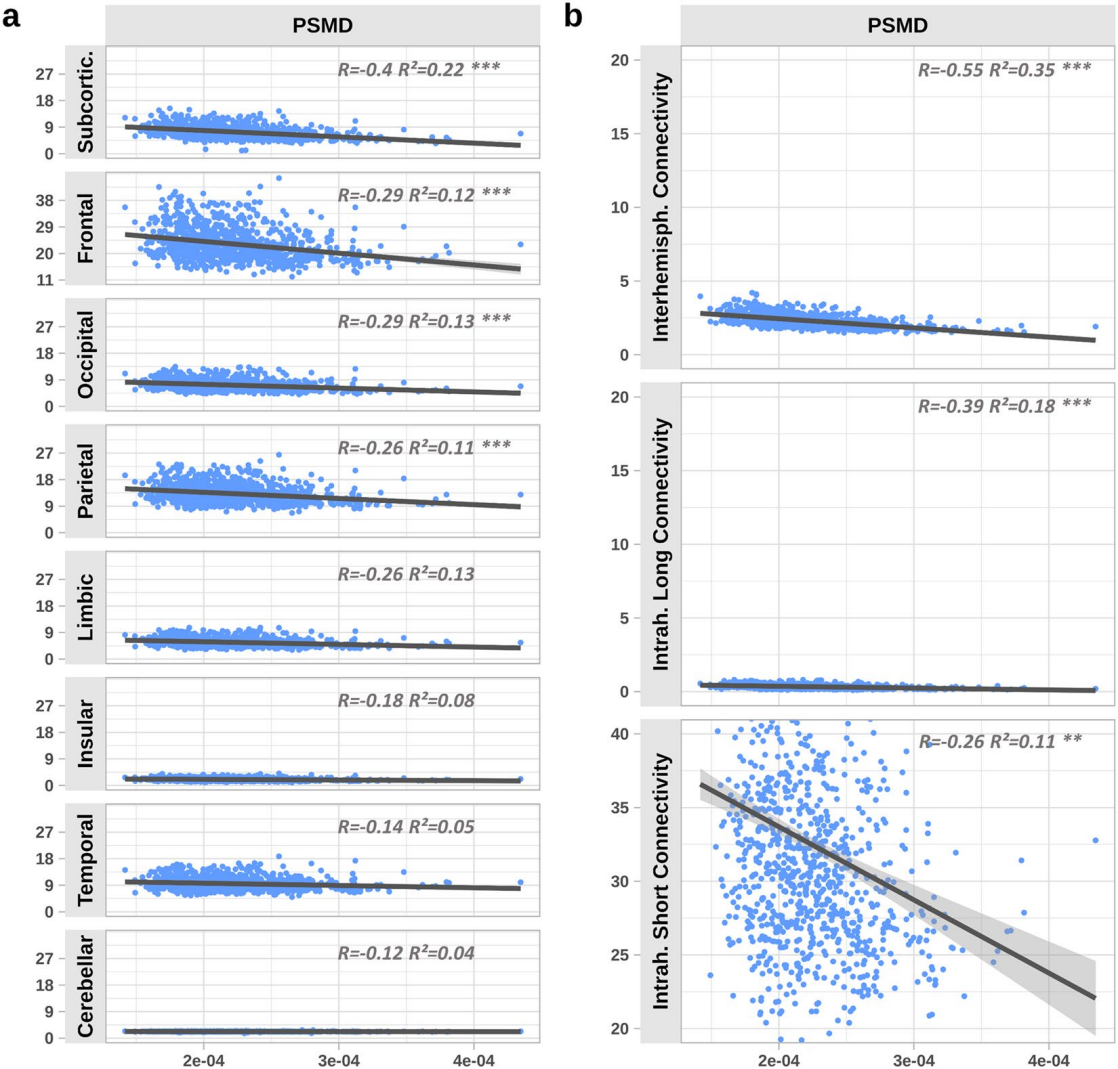
Effects of PSMD on edge groups connected by grey matter region (**a**) and hemispheric course and length (**b**). Simple linear regression results are shown using PSMD as independent and grey matter connectivities as well as course- and length-dependent connectivities as dependent variables. X-axis values correspond to the PSMD (no unit). Y-axis values correspond to the connectivity which represents the sum of all edge weights assigned to a respective group. R corresponds to the linear model before covariate inclusion whereas R^2^ and significance levels (asterisks) correspond to the state after inclusion of covariates. Error bars show the 95% confidence interval. Figures are arranged from highest (top) to lowest (bottom) statistical association. (*p < 0.1; **p < 0.05; ***p < 0.01). Abbreviations: PSMD = peak-width of skeletonised mean diffusivity.

Results from linear models are shown in Fig. 3a. Before covariate inclusion an increased PSMD was associated with a statistically significant decline of connectivity in all groups of edges. Here, the strongest effects were observed between PSMD and subcortical (R = –0.4, p < 0.001), frontal (R = –0.29, p < 0.001) and occipital connectivity (R = –0.29, p < 0.001). On the contrary, insular (R = –0.18, p < 0.001), temporal (R = –0.14, p < 0.01), and cerebellar connectivity (R = –0.12, p < 0.05) were of weakest correlation with PSMD (Supplementary Tables S2). After inclusion of age, sex and brain volume as covariates, correlations involving limbic, insular, temporal and cerebellar connectivity did not remain statistically significant (Supplementary Tables S3). Formal interaction analysis showed that all relationships regarding PSMD and investigated grey matter regions were of statistically significant difference (Supplementary Table S6). Computation of the relative connectivity indicated a preferential association of PSMD with decreased connectivity of subcortical and frontal brain regions: relative subcortical (R = –0.48, p < 0.001), frontal (R = –0.14, p < 0.01) connectivity exhibited negative correlations with PSMD, whereas the relative insular connectivity (R = 0.23, p < 0.001), temporal (R = 0.45, p < 0.001) and cerebellar (R = 0.31, p < 0.001) were positively correlated with PSMD (Supplementary Tables S4 *and* Fig. S1). After covariate inclusion these correlations remained significant (Supplementary Table S5).

#### CSVD effects on intra- or interhemispheric connectivity

The median cut-off to differentiate between long and short edges was 10.37 cm. Figure 3b illustrates the corresponding linear models. Interhemispheric (R = -0.55, p < 0.001), long intrahemispheric (R = –0.39, p < 0.001) and short intrahemispheric (R = –0.26, p < 0.001) edge connectivity significantly decreased with higher PSMD levels (Supplementary Table S2*)*. Results remained significant after covariate inclusion (Supplementary Table S3). Formal interaction analysis showed that all relationships were of statistically significant difference (Supplementary Table S6). Moreover, PSMD correlated negatively with relative interhemispheric (R = –.0.38, p < 0.001) and intrahemispheric long edge connectivity (R = -0.31, p < 0.001), but positively with relative intrahemispheric short edge connectivity (R = 0.43, p < 0.001), thus indicating a specific association of higher PSMD with reduced interhemispheric and long intrahemispheric edge connectivity (Supplementary Table S4 and Fig. S1). These results remained significant after covariate inclusion aswell (Supplementary Table S5). Using WMH instead of PSMD as a predictor, multivariable linear models explained less variance of the observed connectivity data or showed non-significant correlations (Supplementary Tables S7 and S8).

## Discussion

We report on the specific localisation of damage to the structural connectome associated with CSVD in a population-based sample of 930 subjects at increased risk for cerebrovascular diseases. We found that overall, CSVD was associated to a widespread decrease of structural connectivity. However, assessment of the relative connectivity indicated that the observed effects exhibited a regional preference. With regard to specific brain regions, edges connecting subcortical or frontal areas showed the most prominent decrease of connectivity in conjunction with higher CSVD burden as defined by PSMD. Regarding tract course and length, a preferential association of CSVD markers with decreased interhemispheric and long intrahemispheric connection integrity was found. These observations provide insights into structural brain network alterations associated with CSVD and advance the understanding of the pathophysiology of the disease itself and its clinical sequelae.

Effective communication between brain regions is accomplished by white matter pathways. Damage to these white matter pathways resulting from neurological or psychiatric disease hampers communication between brain areas and may further cause secondary damage by remote effects to cortical brain regions^23^. We observed significantly decreased connectivity along with increasing markers of CSVD in a considerable fraction of network edges indicating that a large proportion of white matter fibre pathways are indeed affected by CSVD. Of note, these changes were observed in our group of participants with relatively low WMH volumes compared to previous studies of patients with manifest cognitive impairment or extensive WMH^12,24^. Our findings point to widespread impairment of white matter integrity in the absence of extensive WMH detectable in T2-weighted MRI. Our results further demonstrate that despite of their apparent focal locality in the cerebral white matter, CSVD lesions are linked with disseminated structural changes in the brain architecture. This is in line with the recent perception of CSVD as a global instead of a focal brain disease^6^.

In terms of anatomical localisation, our results showed that edges connecting subcortical or frontal brain regions are more prominently affected in subjects with higher CSVD burden. This was apparent in pronounced significant negative effects regarding subcortical and frontal relative connectivity, whereas in all other groups of edges, relative connectivities were of non-significant or positive association. We hypothesise that specific impairment of frontal and subcortical edges in CSVD are the structural determinants of prominent clinical sequelae such as impaired attention and executive brain functions. In line with our findings, previous studies reported frontal-subcortical pathophysiology in CSVD in terms of reduced glucose metabolism^25^. Lesion symptom inference analysis further indicated that WMH in regions of frontal-subcortical circuits can predict processing speed performance^26^.

CSVD is considered to be among the main contributing factors to vascular cognitive impairment (VCI) and vascular dementia (VaD) which show specific clinical characteristics as compared to other types of dementia. VCI and VaD typically include executive dysfunction, impaired complex information handling and lowered self-control, which can be attributed to dysfunction in frontal brain networks, whereas compared to Alzheimer’s disease the episodic memory deficits seem to be less severe^27–29^. However, how vascular pathology contributes to cognitive decline and dementia remains vague. One hypothesis is that cognitive decline originates from injury to fronto-subcortical pathways^30^. Moreover, it has been hypothesised that cognitive symptoms in CSVD are mediated by frontal atrophy^31,32^. Our findings are coherent with both theories. Frontal subcortical pathways could suffer by preferential frontal and subcortical fibre tract impairment in CSVD. Besides, damaged fibres could induce remote effects like frontal atrophy^14,33^.

In terms of the course of white matter tracts, our observations indicate that interhemispheric and long-range connections are preferentially impaired by CSVD which is in line with findings of previous studies^12,24^. One probable explanation for this is the periventricular predominance of tissue damage in CSVD in close proximity to the course of long-range connections that run in an anterior-posterior trajectory. Accordingly, in our sample WMH, as a common CSVD manifestation, are primarily appearing in periventricular regions.

From a network perspective, interregional communication in the brain is reflected by the connectome’s integrational capacity – i.e., the capability to integrate information from remote regions - which is demonstrably relevant for cognitive function^34,35^. The association between CSVD and cognitive dysfunction was found to be mediated by affection of integrational capacities^12^. Based on our results and the insight from both theoretical models on^36^ and studies of stroke patients^37^, that effective network integration is contingent on the integrity of long-range connections, we suggest that this mediation occurs through the disruptive effect of CSVD on white matter fibre tracts linking remote brain areas. Thus, our findings provide a further piece in the pathophysiological puzzle relating CSVD and cognitive decline.

Besides, functional network investigations indicate that decreased long-range connectivity is associated with Alzheimer’s disease^38^. Conceivably, long-range connectivity is associated with cognitive symptoms across etiologies. Although recent work concludes that VaD and AD are affecting cognitive performance independently^39,40^, our results support the hypothesis of pathophysiological overlap between both entities^41–43^.

### Strengths and limitations of our analysis

Strengths of this work lie in the large sample, and the state-of-the-art and reproducible neuroimaging pipeline. Applying PSMD values as a CSVD surrogate marker taking subvisible changes into account lead to increased explanatory precision in the linear regression analysis compared to the more commonly used measure of absolute WMH volumes.

The limitations are as follows. Although the sample has been risk-enriched by selection of subjects with a certain load of vascular risk factors, it stems from a population-based study and exhibits rather low burden of CSVD-associated lesions visible on structural MRI. Hence, investigations in a more severely affected sample might differ in results.

Since our analysis was focussed on investigating the location of CSVD-induced damage and thus deciphering the association of CSVD with patterns of connectome disruption without relating our findings to behaviour, we can only speculate on possible clinical implications of the observed associations.

Current methods of structural connectome reconstruction are an imperfect attempt to approximate white matter connections in the human brain. Although this methodology offers great advantages for *in-vivo* investigations of structural cerebral connectivity, this general limitation has to be considered when interpreting our results. Diffusion-to-axon mapping, i.e., inferring white matter fibre orientation and integrity from diffusion-weighted imaging, requires assumptions and approximations making it an indirect and error-prone process. A recent study compared state-of-the-art tractography methods by using synthetic data as a gold standard^44^. Accordingly, most approaches reconstruct a considerable amount of false positive streamlines including analysis pipelines very similar to ours. Using our approach, the probability that short streamlines are false positive is higher than for long streamlines. Even modern tractography approaches struggle to reconstruct complex fibre patterns like crossing, kissing, fanning and bending fibres because they lead to similar signal profiles within a voxel^45^. Besides tractography the observed results depend on further analysis design choices like the parcellation scheme underlying the nodes^46^. Our choice is informed by the wide usage of the Desikan-Killiany atlas to provide comparability and the rather coarse scheme increasing robustness of SIFT^47^. Not least, data quality has substantial impact on this type of analysis^46^. Since complex network analysis builds upon the reconstructed connectome, it inherits the shortcomings of the reconstruction approach because it builds upon it. Network characteristics such as global efficiency are abstract concepts that might oversimplify connectional patterns present in large-scale biological systems. However, alterations of large-scale network topology computed by graph theory have shown to be linked to clinical phenotypes of various neurological diseases and therefore present an innovative imaging biomarker in modern structural brain imaging studies^48^. Thus, we encourage the reader to interpret our results with the necessary care.

### Future investigations

While this work limited on a topological approach to locate white matter alterations a fixel-based analysis allows to assess it in 3D space and thus displays a promising complementary approach. Moreover, future work might assess diffusion measurements in a sample not exhibiting any white matter hyperintensities, thus addressing the issue of white matter pathology beyond lesions accessible in T2-weighted MRI.

## Conclusion

We demonstrated that in a sample of participants from a population-based study with an increased risk for vascular disease, white matter damage by CSVD is associated with widespread decreased structural brain connectivity. Specific parts of the structural connectome were preferentially affected. Decreases in connectivity were more pronounced in interhemispheric, long, frontal and subcortical edge groups. Our results link to the supposed pathophysiology of clinical sequelae of CSVD and shed further light on the underlying mechanisms of cognitive and depressive symptoms in CSVD in particular.

## Supplementary information

Supplementary information.

## Data Availability

Anonymised data of the analysis not published within this article will be made available on reasonable request from any qualified investigator after evaluation of the request by the Steering Board of the HCHS.
