# Supplementary material for: Network Localisation of White Matter Damage in Cerebral Small Vessel Disease
Source: Sci Rep. 2020 Jun 8;10:9210. doi: 10.1038/s41598-020-66013-w (PMC7280237; doi:10.1038/s41598-020-66013-w)
Supplement: Supplementary file 1 — Supplementary information. [file 41598_2020_66013_MOESM1_ESM.pdf]

Network Localisation of White Matter Damage in Cerebral Small Vessel Disease – Supplementary Materials

Marvin Petersen[a], Benedikt Frey[a], Eckhard Schlemm[a], Carola Mayer[a], Uta Hanning[b], Kristin Engelke[b], Jens Fiehler[b], Katrin Borof[c], Annika Jagodzinski[c,d], Christian Gerloff[a], Götz Thomalla[a], Bastian Cheng[a]

[a] Department of Neurology, University Medical Center Hamburg-Eppendorf, Hamburg, Germany

[b] Department of Diagnostic and Interventional Neuroradiology, University Medical Center Hamburg-Eppendorf, Hamburg, Germany

[c] Epidemiological study center, University Medical Center Hamburg-Eppendorf, Hamburg, Germany

[d] Department of General and Interventional Cardiology, University Heart and Vascular Center, Hamburg, Germany

Corresponding author:
Marvin Petersen
Department of Neurology
University Medical Center Hamburg-Eppendorf
Martinistraße 52, 20246 Hamburg, Germany

Supplementary text - Image processing

T1w and FLAIR images were both anonymised using pydeface (https://github.com/poldracklab/pydeface) and skull stripped using ANTs' brain extraction script, the former also corrected for field inhomogeneity using the N4 algorithm. Both T1w and FLAIR images were registered rigidly to the b0-image using inverse contrast normalisation.[1] Subsequently, both sequences were used for the grey matter parcellation with the FreeSurfer pipeline. Structural images delineating five tissue types necessary for the anatomically constrained tractography were calculated, whereby voxels containing WMH were tagged as lesioned tissue.[2–5]
Raw diffusion weighted images were denoised by exploiting data redundancy in the PCA domain and Gibbs ringing artefacts were removed based on local subvoxel-shifts.[6,7] For outlier replacement, eddy current and motion correction FSL's eddy was applied with linear second level modelling, B1 field inhomogeneity was estimated using ANTs' N4 algorithm.[8,9] Susceptibility distortion correction has been achieved via non-linear registration using ANTs' SyN: the diffusion weighted image was registered on a T1w image which has been inverted and histogram matched to the b0.[10]
The response functions of the different tissue types were calculated with an unsupervised estimation relying on diffusion data only, fibre orientation distributions (FOD) were estimated subsequently applying single-shell single-tissue constrained spherical deconvolution.[11,12]

Probabilistic Tractography and Streamline weighting
Whole-brain streamlines were reconstructed using anatomically constrained (ACT) probabilistic streamlines tractography by second order integration over fibre orientation distributions (iFOD2), whereby dynamic seeding was applied.[3,13,14] Briefly explained, hereby seeding happens strategically in the white matter to produce streamline bundles that match the distribution of modelled fibre directions in every voxel as well as the reconstructed streamlines density with the fibre density as estimated from the diffusion model. The streamline generation takes into account the uncertainty in the calculation of the fibre orientations and is constrained to anatomical priors to improve the biological plausibility of the tractography. The resulting tractogram was weighted by the 'spherical-deconvolution informed filtering of tractograms 2' (SIFT2) algorithm, whose criteria are adherent to the principles above, i.e. it aims to fit the tractogram to the lobe amplitudes.

Our analysis pipeline will be made public upon publication on our Github page (https://github.com/orgs/csi-hamburg/). Our pipeline design adheres to developer recommendations published on the MRtrix forum (https://community.mrtrix.org/t/wm-odf-and-

response-function-with-dhollander-option---single-shell-versus-multi-shell/572/6; https://community.mrtrix.org/t/distortion-correction-using-t1/975/4).

References:

1. Bhushan, C. *et al.* Co-registration and distortion correction of diffusion and anatomical images based on inverse contrast normalization. *NeuroImage* **115**, 269–280 (2015).
2. Smith, S. M. Fast robust automated brain extraction. *Human Brain Mapping* **17**, 143–155 (2002).
3. Smith, R. E., Tournier, J.-D., Calamante, F. & Connelly, A. Anatomically-constrained tractography: improved diffusion MRI streamlines tractography through effective use of anatomical information. *Neuroimage* **62**, 1924–1938 (2012).
4. Patenaude, B., Smith, S. M., Kennedy, D. N. & Jenkinson, M. A Bayesian model of shape and appearance for subcortical brain segmentation. *Neuroimage* **56**, 907–922 (2011).
5. Zhang, Y., Brady, M. & Smith, S. Segmentation of brain MR images through a hidden Markov random field model and the expectation-maximization algorithm. *IEEE Trans Med Imaging* **20**, 45–57 (2001).
6. Veraart, J. *et al.* Denoising of diffusion MRI using random matrix theory. *Neuroimage* **142**, 394–406 (2016).
7. Kellner, E., Dhital, B., Kiselev, V. G. & Reisert, M. Gibbs-ringing artifact removal based on local subvoxel-shifts. *Magn Reson Med* **76**, 1574–1581 (2016).
8. Andersson, J. L. R. & Sotiropoulos, S. N. An integrated approach to correction for off-resonance effects and subject movement in diffusion MR imaging. *Neuroimage* **125**, 1063–1078 (2016).
9. Tustison, N. J. *et al.* N4ITK: Improved N3 Bias Correction. *IEEE Transactions on Medical Imaging* **29**, 1310–1320 (2010).
10. Avants, B., Epstein, C., Grossman, M. & Gee, J. Symmetric diffeomorphic image registration with cross-correlation: Evaluating automated labeling of elderly and neurodegenerative brain. *Medical Image Analysis* **12**, 26–41 (2008).

11. Jeurissen, B., Tournier, J.-D., Dhollander, T., Connelly, A. & Sijbers, J. Multi-tissue constrained spherical deconvolution for improved analysis of multi-shell diffusion MRI data. *NeuroImage* **103**, 411–426 (2014).

12. Tournier, J.-D., Calamante, F., Gadian, D. G. & Connelly, A. Direct estimation of the fiber orientation density function from diffusion-weighted MRI data using spherical deconvolution. *NeuroImage* **23**, 1176–1185 (2004).

13. Tournier, J.-D., Calamante, F. & Connelly, A. Improved probabilistic streamlines tractography by 2. order integration over fibre orientation distributions. **88**, 2010 (2010).

14. Smith, R. E., Tournier, J.-D., Calamante, F. & Connelly, A. SIFT2: Enabling dense quantitative assessment of brain white matter connectivity using streamlines tractography. *NeuroImage* **119**, 338–351 (2015).

Supplementary table S1 - Desikan areas ordered by assigned gray matter division

Divisions	Desikan areas
Frontal nodes	ctx-lh-caudalmiddlefrontal, ctx-rh-caudalmiddlefrontal, ctx-lh-lateralorbitofrontal, ctx-rh-lateralorbitofrontal, ctx-lh-medialorbitofrontal, ctx-rh-medialorbitofrontal, ctx-lh-paracentral, ctx-rh-paracentral, ctx-lh-parsopercularis, ctx-rh-parsopercularis, ctx-lh-parsorbitalis, ctx-rh-parsorbitalis, ctx-lh-parstriangularis, ctx-rh-parstriangularis, ctx-lh-precentral, ctx-rh-precentral, ctx-lh-rostralmiddlefrontal, ctx-rh-rostralmiddlefrontal, ctx-lh-superiorfrontal, ctx-rh-superiorfrontal, ctx-lh-frontalpole, ctx-rh-frontalpole
Temporal nodes	ctx-lh-bankssts, ctx-rh-bankssts, ctx-lh-entorhinal, ctx-rh-entorhinal, ctx-lh-fusiform, ctx-rh-fusiform, ctx-lh-inferiortemporal, ctx-rh-inferiortemporal, ctx-lh-middletemporal, ctx-rh-middletemporal, ctx-lh-superiortemporal, ctx-rh-superiortemporal, ctx-lh-transversetemporal, ctx-rh-transversetemporal, ctx-lh-temporalpole, ctx-rh-temporalpole
Parietal nodes	ctx-lh-inferiorparietal, ctx-rh-inferiorparietal, ctx-lh-postcentral, ctx-rh-postcentral, ctx-lh-precuneus, ctx-rh-precuneus, ctx-lh-superiorparietal, ctx-rh-superiorparietal, ctx-lh-supramarginal, ctx-rh-supramarginal
Occipital nodes	ctx-lh-lateraloccipital, ctx-rh-lateraloccipital, ctx-lh-lingual, ctx-rh-lingual, , ctx-lh-cuneus, ctx-rh-cuneus, ctx-lh-pericalcarine, ctx-rh-pericalcarine

Insular nodes	ctx-lh-insula, ctx-rh-insula
Cerebellar nodes	Left-Cerebellar-Cortex, Right-Cerebellar-Cortex
Limbic nodes	ctx-lh-caudalanteriorcingulate, ctx-rh-caudalanteriorcingulate, ctx-lh-isthmuscingulate, ctx-rh-isthmuscingulate, ctx-lh-posteriorcingulate, ctx-rh-posteriorcingulate, ctx-lh-rostralanteriorcingulate, ctx-rh-rostralanteriorcingulate, Left-Hippocampus, Right-Hippocampus, Left-Amygdala, Right-Amygdala, ctx-lh-parahippocampal, ctx-rh-parahippocampal
Subcortical nodes	Left-Thalamus-Proper, Right-Thalamus-Proper, Left-Caudate, Right-Caudate, Left-Putamen, Right-Putamen, Left-Pallidum, Right-Pallidum, Left-Accumbens-area, Right-Accumbens-area

Supplementary tables S2 - Simple linear models - Connectivity ~ PSMD

Linear models - Connectivity ~ PSMD

	Dependent variable:										
	long	short	interhem	frontal	temporal	parietal	occipital	limbic	insular	subcortical	cerebellar
psmd	-1,235.600***	-49,635.140***	-6,248.118***	-39,940.240***	-7,767.961***	-20,911.020***	-12,784.290***	-8,963.120***	-2,223.154***	-21,399.450***	-248.486***
	-95.908	-6,025.70	-308.36	-4,335.68	-1,741.50	-2,531.92	-1,380.38	-1,097.70	-388.277	-1,588.73	-66.207
Constant	0.612***	43.621***	3.692***	32.082***	11.253***	17.887***	10.027***	7.668***	2.511***	12.126***	2.298***
	-0.022	-1.365	-0.07	-0.982	-0.394	-0.574	-0.313	-0.249	-0.088	-0.36	-0.015
Observations	930	930	930	930	930	930	930	930	930	930	930
R^2	0.152	0.068	0.307	0.084	0.021	0.068	0.085	0.067	0.034	0.164	0.015
Adjusted R^2	0.151	0.067	0.306	0.083	0.02	0.067	0.084	0.066	0.033	0.163	0.014
Residual Std. Error (df = 928)	0.115	7.201	0.369	5.182	2.081	3.026	1.65	1.312	0.464	1.899	0.079
F Statistic (df = 1; 928)	165.975***	67.852***	410.566***	84.861***	19.896***	68.210***	85.774***	66.673***	32.784***	181.428***	14.086***
Note:											*p<0.1; **p<0.05; ***p<0.01

# p-Values after Bonferroni Correction

**p-Values: Connectivity ~ PSMD**	
Dependent variable	PSMD
Long edge connectivity	9.21E-33
Short edge connectivity	1.18E-13
Interhem. edge connectivity	1.42E-73
Frontal edge connectivity	4.12E-17
Temporal edge connectivity	0.001817
Parietal edge connectivity	9.96E-14
Occipital edge connectivity	2.70E-17
Limbic edge connectivity	2.06E-13
Insular edge connectivity	2.75E-06
Subcortical edge connectivity	1.32E-35
Cerebellar edge connectivity	0.036721

Supplementary table S3 - Linear models - Absolute connectivity ~ PSMD + Covariates

Linear models - Connectivity ~ PSMD + Age + Sex + Brain volume

	Dependent variable:										
	long	short	interhem	frontal	temporal	parietal	occipital	limbic	insular	subcortical	cerebellar
psmd	-851.190***	-26,611.330***	-4,995.058***	-24,856.050***	-1,816.74	-12,425.110***	-7,582.786***	-3,576.26	-825.003	-13,599.550***	-233.667
	-112.688	-7,067.76	-358.146	-5,095.95	-2,056.56	-2,976.11	-1,620.88	-1,271.21	-455.872	-1,843.12	-78.554
age	-0.003***	-0.168***	-0.008***	-0.107***	-0.046***	-0.060***	-0.037***	-0.040***	-0.010***	-0.060***	0.0002
	-0.001	-0.034	-0.002	-0.024	-0.01	-0.014	-0.008	-0.006	-0.002	-0.009	-0.0004
sex	0.026	2.387**	0.173***	1.816***	0.477	1.101***	0.511**	0.480***	0.161***	0.599**	0.028***
	-0.009	-0.586	-0.03	-0.423	-0.171	-0.247	-0.134	-0.105	-0.038	-0.153	-0.007
brain volume	0	0	0	0	0	0	0	0	0	0	0.00000***
	0	0	0	0	0	0	0	0	0	0	0
Constant	0.662***	40.736***	3.433***	28.680***	11.177***	15.173***	10.189***	7.818***	2.196***	12.332***	2.098***
	-0.07	-4.394	-0.223	-3.168	-1.278	-1.85	-1.008	-0.79	-0.283	-1.146	-0.049
Observations	930	930	930	930	930	930	930	930	930	930	930
R^2	0.188	0.111	0.352	0.122	0.053	0.108	0.125	0.132	0.077	0.219	0.039
Adjusted R^2	0.185	0.107	0.349	0.119	0.049	0.104	0.121	0.129	0.073	0.216	0.034
Residual Std. Error (df = 925)	0.112	7.045	0.357	5.079	2.05	2.966	1.616	1.267	0.454	1.837	0.078
F Statistic (df = 4; 925)	53.551***	28.898***	125.372***	32.254***	13.035***	27.885***	32.995***	35.306***	19.243***	65.003***	9.263***
Note:											*p<0.1; **p<0.05; ***p<0.01

p-Values after Bonferroni Correction

p-Values: Connectivity ~ PSMD + Age + Sex + Brain Volume				
Dependent variable	PSMD	Age	Sex	Brain Volume
Long edge connectivity	2.01E-11	1.02E-05	0.97544	1
Short edge connectivity	0.03503412	0.000148	0.010031	1
Interhem. edge connectivity	5.71E-38	0.000368	1.48E-06	1
Frontal edge connectivity	0.00025023	0.00256	0.003808	1
Temporal edge connectivity	1	0.000716	1	1
Parietal edge connectivity	0.00645883	0.005875	0.001814	1
Occipital edge connectivity	0.00065849	0.000414	0.030327	1
Limbic edge connectivity	0.99154938	2.14E-08	0.001191	1
Insular edge connectivity	1	0.000815	0.004467	1
Subcortical edge connectivity	7.05E-11	3.28E-09	0.019031	1
Cerebellar edge connectivity	0.5959262	1	0.002946	0.007883784

Supplementary tables S4 - Simple linear models – Relative connectivity ~ PSMD

Linear models - Relative Connectivity ~ PSMD

	Dependent variable:										
	rel_long	rel_short	rel_interhem	rel_frontal	rel_temporal	rel_parietal	rel_occipital	rel_limbic	rel_insular	rel_subcortical	rel_cerebellar
	-1	-2	-3	-4	-5	-6	-7	-8	-9	-10	-11
psmd	-20.203***	93.805***	-73.602***	-36.009***	117.560***	6.577	-19.434	4.551	16.041***	-139.120***	49.835***
	-2.034	-6.511	-5.95	-8.302	-7.593	-7.14	-6.172	-4.032	-2.201	-8.239	-5.065
Constant	0.014***	0.903***	0.083***	0.337***	0.110***	0.186***	0.106***	0.079***	0.025***	0.135***	0.022***
	-0.0005	-0.001	-0.001	-0.002	-0.002	-0.002	-0.001	-0.001	-0.0005	-0.002	-0.001
Observations	930	930	930	930	930	930	930	930	930	930	930
R^2	0.096	0.183	0.142	0.02	0.205	0.001	0.011	0.001	0.054	0.235	0.094
Adjusted R^2	0.095	0.182	0.141	0.019	0.204	-0.0002	0.01	0.0003	0.053	0.234	0.093
Residual Std. Error (df = 928)	0.002	0.008	0.007	0.01	0.009	0.009	0.007	0.005	0.003	0.01	0.006
F Statistic (df = 1; 928)	98.692***	207.598***	153.002***	18.811***	239.719***	0.848	9.914***	1.274	53.124***	285.119***	96.789***
Note:											$^{*}p<0.1$; $^{**}p<0.05$; $^{***}p<0.01$

p-Values after Bonferroni Correction

**p-Values: Relative Connectivity ~ PSMD**	
Dependent variable	PSMD
Rel. long edge connectivity	7.13E-20
Rel. short edge connectivity	2.51E-40
Rel. interhem. edge connectivity	2.42E-30
Rel. frontal edge connectivity	0.003171
Rel. temporal edge connectivity	5.68E-46
Rel. parietal edge connectivity	1
Rel. occipital edge connectivity	0.335189
Rel. limbic edge connectivity	1
Rel. insular edge connectivity	1.33E-10
Rel. subcortical edge connectivity	1.09E-53
Rel. cerebellar edge connectivity	1.70E-19

Supplementary table S5 - Linear models - Relative connectivity ~ PSMD + covariates

Linear models - Relative Connectivity ~ PSMD + Age + Sex + Brain volume

	Dependent variable:										
	rel_long	rel_short	rel_interhem	rel_frontal	rel_temporal	rel_parietal	rel_occipital	rel_limbic	rel_insular	rel_subcortical	rel_cerebellar
psmd	-15.854***	98.751***	-82.898***	-50.762***	104.936***	-2.435	-18.988	23.564***	16.090***	-101.030***	28.626***
	-2.428	-7.791	-7.102	-9.891	-8.99	-8.49	-7.357	-4.691	-2.641	-9.621	-5.918
age	-0.00004	-0.0001	0.0001	0.0001	0.0001	0.0001	0.00001	-0.0001***	0	-0.0003***	0.0002***
	-0.00001	-0.00004	-0.00003	-0.00005	-0.00004	-0.00004	-0.00004	-0.00002	-0.00001	-0.00005	-0.00003
sex	0.00003	-0.0001	0.0001	0.002	-0.004***	0.002	-0.0002	0.001	0.0002	0.001	-0.002***
	-0.0002	-0.001	-0.001	-0.001	-0.001	-0.001	-0.001	-0.0004	-0.0002	-0.001	-0.0005
brain volume	0	0	0	0	0	0.000**	0	0	0	0	0
	0	0	0	0	0	0	0	0	0	0	0
Constant	0.016***	0.901***	0.082***	0.317***	0.123***	0.164***	0.116***	0.088***	0.023***	0.144***	0.024***
	-0.002	-0.005	-0.004	-0.006	-0.006	-0.005	-0.005	-0.003	-0.002	-0.006	-0.004
Observations	930	930	930	930	930	930	930	930	930	930	930
R^2	0.107	0.189	0.152	0.035	0.227	0.021	0.025	0.063	0.056	0.277	0.143
Adjusted R^2	0.103	0.185	0.148	0.031	0.224	0.017	0.021	0.059	0.052	0.274	0.139
Residual Std. Error (df = 925)	0.002	0.008	0.007	0.01	0.009	0.008	0.007	0.005	0.003	0.01	0.006
F Statistic (df = 4; 925)	27.619***	53.781***	41.463***	8.489***	68.096***	4.897***	6.031***	15.494***	13.647***	88.505***	38.575***
Note:											*p<0.1; **p<0.05; ***p<0.01

p-Values after Bonferroni Correction

**p-Values: Relative Connectivity ~ PSMD + Age + Sex + Brain Volume**				
Dependent variable	PSMD	Age	Sex	Brain Volume
Rel. long edge connectivity	2.15E-08	0.327405	1	1
Rel. short edge connectivity	9.06E-32	1	1	1
Rel. interhem. edge connectivity	3.63E-27	1	1	1
Rel. frontal edge connectivity	6.92E-05	0.344704	1	1
Rel. temporal edge connectivity	3.64E-27	1	0.000303	1
Rel. parietal edge connectivity	1	1	0.688836	0.02266092
Rel. occipital edge connectivity	1	1	1	0.363577302
Rel. limbic edge connectivity	0.00012097	2.06E-08	1	1
Rel. insular edge connectivity	3.22E-07	1	1	1
Rel. subcortical edge connectivity	3.75E-22	7.58E-10	1	1
Rel. cerebellar edge connectivity	0.00030562	5.75E-06	0.006801	1

Supplementary tables S6 – *Formal* interaction analysis

**Interaction analysis<br>Absol. Connectivity ~ PSMD * Gray matter region + (1|Subject)**		
Term	Estimate	Std. Error
Intercept	-2.298e-14	2.957e-02
PSMD	-1.223e-01***	2.957e-02
psmd:regionfrontal	-1.672e-01***	1.138e-02
psmd:regiontemporal	-2.260e-02**	1.138e-02
psmd:regionparietal	-1.394e-01***	1.138e-02
psmd:regionoccipital	-1.686e-01***	1.138e-02
psmd:regionsubcortical	-2.821e-01***	1.138e-02
psmd:regionlimbic	-1.366e-01***	1.138e-02
psmd:regioninsular	-6.244e-01***	1.138e-02
Note: *p<0.1; **p<0.05; ***p<0.01		

**Interaction analysis<br>Absol. Connectivity ~ PSMD * Fibre length + (1|Subject)**		
Term	Estimate	Std. Error
Intercept	-2.65e-14	0.0269
PSMD	-5.54e-11***	0.0269
psmd:lengthlong	1.70e-01***	0.0687
psmd:lengtshort	2.92e-01***	0.0687
Note: *p<0.1; **p<0.05; ***p<0.01		

Supplementary tables S7 - Linear models - Absolute connectivity ~ WMH load

Linear models - Connectivity ~ Log WMH load + Age + Sex + Brain volume

	Dependent variable:										
	long	short	interhem	frontal	temporal	parietal	occipital	limbic	insular	subcortical	cerebellar
wmh load log	-0.013	-0.309	-0.057***	-0.309	0.064	-0.173	-0.108	-0.051	0.001	-0.18	-0.003
	-0.004	-0.264	-0.015	-0.191	-0.076	-0.111	-0.061	-0.047	-0.017	-0.07	-0.003
age	-0.004***	-0.213***	-0.017***	-0.148***	-0.055***	-0.079***	-0.048***	-0.045***	-0.012***	-0.082***	-0.0002
	-0.001	-0.034	-0.002	-0.025	-0.01	-0.015	-0.008	-0.006	-0.002	-0.009	-0.0004
sex	0.036**	2.654***	0.223***	2.069***	0.48	1.231***	0.591***	0.518***	0.167***	0.739***	0.031***
	-0.01	-0.589	-0.032	-0.427	-0.17	-0.248	-0.136	-0.106	-0.038	-0.157	-0.007
brain volume	0	0	0	0	0	0	0	0	0	0	0.00000***
	0	0	0	0	0	0	0	0	0	0	0
Constant	0.572***	38.364***	2.992***	26.384***	11.353***	13.952***	9.435***	7.463***	2.164***	11.031***	2.075***
	-0.074	-4.535	-0.249	-3.284	-1.311	-1.912	-1.043	-0.813	-0.291	-1.204	-0.05
Observations	930	930	930	930	930	930	930	930	930	930	930
R^2	0.147	0.099	0.228	0.102	0.053	0.093	0.107	0.126	0.074	0.179	0.031
Adjusted R^2	0.143	0.095	0.225	0.098	0.049	0.089	0.103	0.122	0.07	0.176	0.027
Residual Std. Error (df = 925)	0.115	7.093	0.39	5.137	2.05	2.99	1.632	1.272	0.455	1.884	0.079
F Statistic (df = 4; 925)	39.872***	25.349***	68.313***	26.370***	13.017***	23.753***	27.761***	33.372***	18.360***	50.531***	7.330***

Note: $^{*}p<0.1$; $^{**}p<0.05$; $^{***}p<0.01$

p-Values after Covariate Inclusion and Bonferroni Correction

**p-Values: Connectivity ~ WMH load + Age + Sex + Brain Volume**				
Dependent variable	WMH load	Age	Sex	Brain Volume
Long edge connectivity	0.34008152	5.46E-11	0.0426	1
Short edge connectivity	1	1.72E-07	0.001497	1
Interhem. edge connectivity	0.01853704	7.37E-16	1.93E-09	1
Frontal edge connectivity	1	9.31E-07	0.000291	1
Temporal edge connectivity	1	8.69E-06	0.983001	1
Parietal edge connectivity	1	1.49E-05	0.000171	1
Occipital edge connectivity	1	2.81E-07	0.002909	1
Limbic edge connectivity	1	1.25E-10	0.000227	1
Insular edge connectivity	1	8.04E-06	0.002111	1
Subcortical edge connectivity	1	5.08E-16	0.000529	1
Cerebellar edge connectivity	1	1	0.000539	0.01423747

Supplementary tables S8 - Linear models - Relative connectivity ~ WMH load

Linear models - Relative Connectivity ~ Log WMH load + Age + Sex + Brain volume

	Dependent variable:										
	rel_long	rel_short	rel_interhem	rel_frontal	rel_temporal	rel_parietal	rel_occipital	rel_limbic	rel_insular	rel_subcortical	rel_cerebellar
wmh load log	-0.0003	0.001**	-0.001	-0.001	0.002***	-0.0004	-0.001	0.0002	0.0004**	-0.002***	0.0004
	-0.0001	-0.0003	-0.0003	-0.0004	-0.0003	-0.0003	-0.0003	-0.0002	-0.0001	-0.0004	-0.0002
age	-0.0001***	0.0001	-0.0001	0.0001	0.0001	0.0001	0	-0.0001***	0.00001	-0.0005***	0.0002***
	-0.00001	-0.00004	-0.00004	-0.00005	-0.00005	-0.00004	-0.00004	-0.00002	-0.00001	-0.00005	-0.00003
sex	0.0002	-0.001	0.001	0.002	-0.005***	0.002	0.00003	0.001	-0.00004	0.002	-0.002***
	-0.0002	-0.001	-0.001	-0.001	-0.001	-0.001	-0.001	-0.0004	-0.0002	-0.001	-0.0005
brain volume	0	0	0	0	0	0.000***	-0.000***	0	0	0	0
	0	0	0	0	0	0	0	0	0	0	0
Constant	0.015***	0.910***	0.075***	0.311***	0.137***	0.163***	0.113***	0.090***	0.026***	0.133***	0.027***
	-0.002	-0.005	-0.005	-0.006	-0.006	-0.005	-0.005	-0.003	-0.002	-0.006	-0.004
Observations	930	930	930	930	930	930	930	930	930	930	930
R^2	0.076	0.063	0.038	0.014	0.159	0.023	0.022	0.038	0.033	0.205	0.125
Adjusted R^2	0.072	0.059	0.034	0.01	0.155	0.018	0.018	0.034	0.029	0.202	0.121
Residual Std. Error (df = 925)	0.002	0.008	0.008	0.01	0.009	0.008	0.007	0.005	0.003	0.01	0.006
F Statistic (df = 4; 925)	19.063***	15.463***	9.054***	3.330**	43.585***	5.347***	5.282***	9.171***	7.968***	59.757***	32.902***
Note:											*p<0.1; **p<0.05; ***p<0.01

p-Values after Covariate Inclusion and Bonferroni Correction

Dependent variable	WMH load	Age	Sex	Brain Volume
Rel. long edge connectivity	0.22586364	0.00083	1	1
Rel. short edge connectivity	0.02633456	1	1	1
Rel. interhem. edge connectivity	0.29963391	1	1	1
Rel. frontal edge connectivity	1	1	1	1
Rel. temporal edge connectivity	8.25E-10	0.261248	1.01E-07	1
Rel. parietal edge connectivity	1	0.656669	0.431719	0.015534867
Rel. occipital edge connectivity	1	1	1	0.338477152
Rel. limbic edge connectivity	1	0.003928	1	1
Rel. insular edge connectivity	0.025202	1	1	1
Rel. subcortical edge connectivity	0.00727283	1.61E-18	1	1
Rel. cerebellar edge connectivity	1	1.42E-09	0.000503	1

Supplementary figure S1 - Linear models - Relative connectivity ~ PSMD

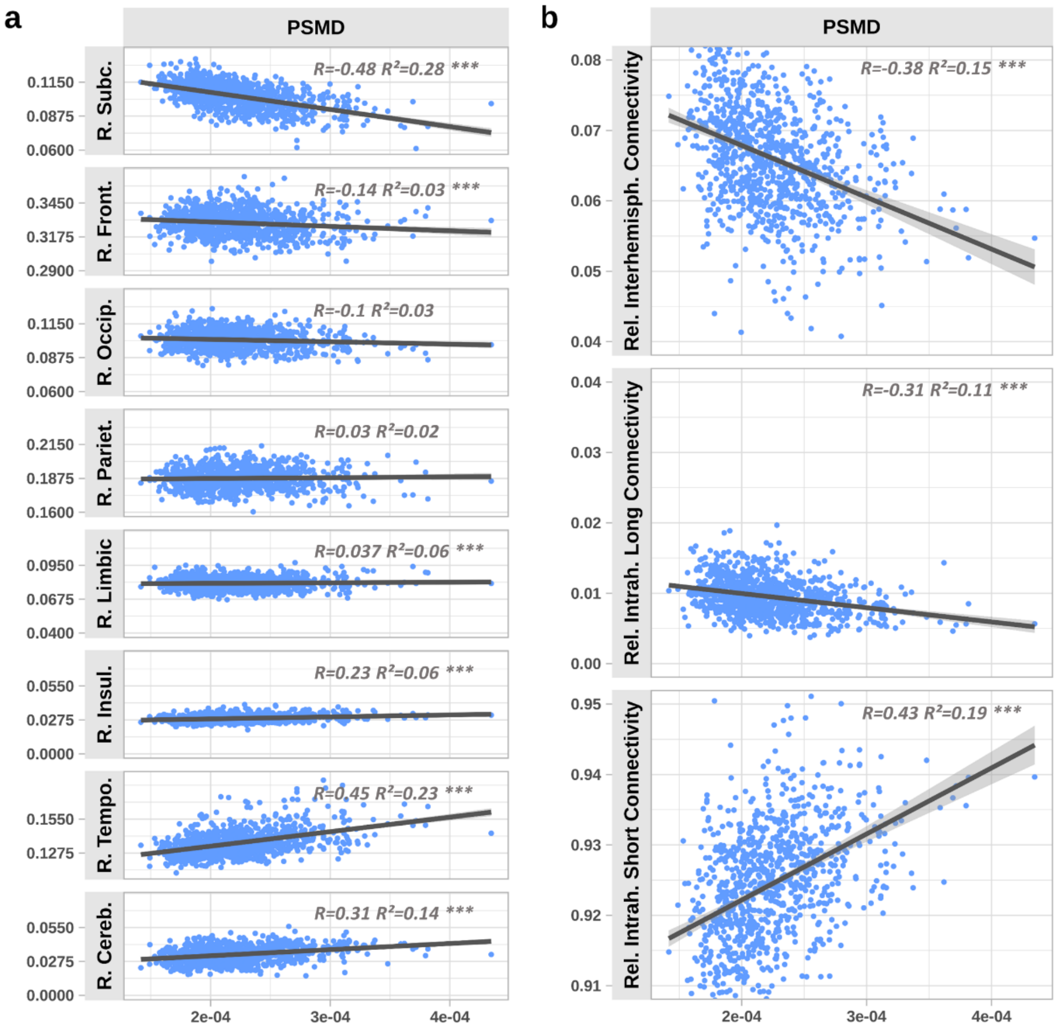

Effects of PSMD on relative connectivities of edges grouped by grey matter region (**a**) and hemispheric course and length (**b**). The relative connectivity represents the share of the respective edge-group connectivity in the connectivity of all groups totalled. The relative connectivity allows assessment of which edge groups change to a greater extent: if the share of an edge group on the total connectivity shrinks with higher CSVD burden we conclude it is preferentially affected. Per grouping scheme, the relative edge group connectivities add up to the total connectivity, the relative connectivities to one. Simple linear regression results are shown using PSMD (no unit) as independent and relative grey matter connectivities as well as course- and length-dependent connectivities as dependent variables. R corresponds to the linear model before covariate inclusion whereas R^2 and significance levels (asterisks) correspond to the state after inclusion of covariates. Error bars show the 95% confidence

interval. (*p<0.1; **p<0.05; ***p<0.01) Abbreviations: PSMD: Peak-width of skeletonised mean diffusivity
